# Oral Administration of *Lactobacillus acidophilus* LA5 Prevents Alveolar Bone Loss and Alters Oral and Gut Microbiomes in a Murine Periodontitis Experimental Model

**DOI:** 10.3390/microorganisms12061057

**Published:** 2024-05-24

**Authors:** Amalia C. S. Cataruci, Dione Kawamoto, Natali Shimabukuro, Karin H. Ishikawa, Ellen S. Ando-Suguimoto, Rodolfo A. Ribeiro, Gianlucca G. Nicastro, Emanuel Albuquerque-Souza, Robson F. de Souza, Marcia P. A. Mayer

**Affiliations:** 1 Department of Microbiology, Institute of Biomedical Sciences, University of São Paulo, São Paulo 05508-220, SP, Brazil; acs.cataruci@gmail.com (A.C.S.C.); 77didi@gmail.com (D.K.); natali.shimabukuro@hotmail.com (N.S.); karinhitomi@hotmail.com (K.H.I.); rodolfo.alvarenga.ribeiro@usp.br (R.A.R.); ggnicastro@gmail.com (G.G.N.); emmanuel.desouza@qmul.ac.uk (E.A.-S.); rfsouza@usp.br (R.F.d.S.); mpamayer@icb.usp.br (M.P.A.M.); 2 Division of Periodontics, Department of Stomatology, School of Dentistry, University of São Paulo, São Paulo 05508-220, SP, Brazil; 3 William Harvey Research Institute, Queen Mary University of London, London E1 2AT, UK

**Keywords:** *Lactobacillus*, periodontitis, probiotics

## Abstract

Periodontitis is a destructive inflammatory response triggered by dysbiosis. *Lactobacillus acidophilus* LA5 (LA5) may impair microbial colonization and alter the host. Thus, we evaluated the effect of LA5 on alveolar bone loss in a periodontitis murine model and investigated its effect on the oral and gut microbiomes. *Porphyromonas gingivalis, Prevotella intermedia, Fusobacterium nucleatum,* and *Streptococcus gordonii* were inoculated in C57BL/6 mice (P+), with LA5 (L+). SHAM infected controls (P- and/or L- groups) were also evaluated. After 45 days, alveolar bone loss in the maxilla and oral and gut microbiomes were determined. The administration of LA5 controlled the microbial consortium-induced alveolar bone loss. Periodontopathogens infection resulted in shifts in the oral and gut microbiomes consistent with dysbiosis, and LA5 reshaped these changes. The oral microbiome of P+L- group showed the increased abundance of *Enterococaccea, Streptoccocaceae*, *Staphylococcaceae*, *Moraxellaceae*, and *Pseudomonadaceae*, which were attenuated by the administration of LA5 to the infected group (P+L+). The administration of LA5 to otherwise non-infected mice resulted in the increased abundance of the superphylum Patescibacteria and the family *Saccharamonadaceae* in the gut. These data indicate *L. acidophilus* LA5 as a candidate probiotic for the control of periodontitis.

## 1. Introduction

Periodontitis is a destructive inflammatory disease triggered by a dysbiotic oral microbiome [1], which is also followed by an altered gut microbial composition, as evidenced by murine and human studies [2,3,4]. The administration of probiotics, i.e., live bacteria that provide benefits to health [5], seems a promising approach to reestablishing a balanced microbiome and control periodontitis [6]. The benefits of controlling periodontitis progression by the use of probiotics may be extended to other inflammatory disorders, such as diabetes and cardiovascular diseases [7], and the search for a strain that is efficient in controlling the dysbiosis underlying these conditions is of paramount importance for the long-term benefit of probiotic therapy [8].

Probiotic lactobacilli can produce direct antimicrobial compounds [6] and modulate the host response [9]. Their administration reduced the number of residual pockets in periodontitis-treated patients [10] and decreased the levels of pathogenic bacteria in saliva and subgingival biofilm [11]. Meta-analyses studies reported that the administration of certain lactobacilli probiotics is able to control gingivitis and periodontitis [12,13]. However, different probiotic strains and regimens were used, complicating the comparison among studies.

The concept for the use of probiotics to control periodontitis involves their ability to decrease oral colonization by pathogens without affecting commensal organisms, and to modulate the inflammatory response. By controlling inflammation, probiotics also alter resident microbial communities.

The strain *L. acidophilus* LA5 is safe to humans and has been successfully used in the control of gestational hyperglycemia [14], glycemic control in patients with metabolic syndrome [15], control of systemic inflammation in obese people [16], and improvement in several parameters such as hepatic transaminases, serum total cholesterol, and low-density lipoprotein cholesterol levels [17], evidencing its potential to control inflammatory diseases.

Our previous in vitro data revealed that *L. acidophilus* LA5 was able to inhibit the adhesion and invasion of periodontopathogens to gingival epithelial cells [18,19] and reduced *Porphyromonas gingivalis* abundance in multispecies biofilms without interfering in commensals [20]. Moreover, secreted products of *L. acidophilus* LA5 inhibited biofilm formation and affected pre-formed biofilms of *Aggregatibacter actinomycetemcomitans* [21]. In fact, *L. acidophilus* LA5 and its by-products inhibited the transcription of *P. gingivalis* and *A. actinomycetemcomitans* key virulence factors [20,21]. Our screening studies aiming to determine probiotics strains suitable for the control of periodontitis also highlighted LA5 due to its ability to attenuate the production of pro-inflammatory cytokines by *P. gingivalis* or *A. actinomycetemcomitans*-infected gingival epithelial cells [18,19]. Furthermore, LA5 was able to control alveolar bone loss in a periodontitis mice model induced by *Agreggatibacter actinomycetemcomitans* and *Streptococcus gordonii* [22].

The clinical use of probiotics is dependent on the apprehension of their properties, in order to select the most appropriate agent for each condition [23]. In vitro analyses are only the first step of pre-clinical studies, and the effect of a probiotic strain must be determined experimentally in vivo. Aiming to further assess the potential of *L. acidophilus* LA5 to control periodontitis, we evaluated its ability to control alveolar bone loss and modulate the oral and gut microbiomes in a murine periodontitis model.

## 2. Materials and Methods

### 2.1. Animals and Group Allocation

Four-week-old C57Bl6 mice bred under specific pathogen-free conditions were purchased from the mouse breeding facility of the Medical School, University of São Paulo. All procedures were performed in accordance with the ARRIVE 2.0 guidelines and with the approval of the Institutional Animal Care and Use Committee (Approval number: CEUA/ICB-USP protocol 111; CEUA/FO-USP protocol 20/2019).

Mice presenting alterations in growth, physical defects, and/or weight were excluded at baseline. After one week of acclimation at the animal facility of the Department of Microbiology, Institute of Biomedical Sciences, University of São Paulo, the resident microbiome was reduced by the use of antimicrobials. Then, animals were randomly allocated into four groups: the periodontitis group (P+L-) received a microbial consortium formed by human periodontopathogens; the test group (P+L+) was treated with the pathogenic consortium plus *L. acidophilus* LA5; the probiotic group (P-L+) received only *L. acidophilus* LA5; and the SHAM infected group (P-L-) received the vehicles. The animals were followed for 45 days, counting from the first microbial inoculation, and monitored for weight, mobility, fur, and skin lesions throughout the experimental period.

Each animal was assigned a temporary random number within the group, and a cage was selected randomly from the pool of all cages and given a numerical designation. During the allocation, the assessment of the results and data analysis blinding was performed. However, blindness was not possible during the inoculation of microorganisms, since the same researcher prepared the suspensions, and these bacterial culture suspensions differed in color from the vehicle.

### 2.2. Sample Size

Sample calculation was performed based on previous data, using alveolar bone loss as the primary outcome [24]. Taking into consideration the difference in the bone volume of 4719 cubic pixels at a standard area, a sample size of 7.84 animals was adequate to obtain a Type I error rate of 5% and power greater than 80% [25]. Thus, each experimental group consisted of eight animals.

### 2.3. Bacteria Cultures

*Lactobacillus acidophilus* LA-5™ (CHR Hansen Holding A/S, Hørsholm, Denmark) was grown in MRS broth (BD; Becton, Dickinson and Company, Sparks, NV, USA). The microbial consortium for inducing experimental periodontitis was produced as described previously [24]. Briefly, the oral organisms of human origin *P. gingivalis* ATCC 33,277 (non-capsulated fimbriated fimA I), *P. gingivalis* (W83 encapsulated K1, afimbriated), *Prevotella intermedia 17* [26], and *F. nucleatum* ATCC 25586 [27] were cultivated in an anaerobic chamber (85% N_2_, 5% H_2_, and 10% CO_2_) in BHI HM [brain heart infusion broth (BD) supplemented with yeast extract, hemin (1 mg/mL; Sigma-Aldrich, Darmstadt, Germany), and menadione (0.1 mg/mL; Sigma-Aldrich)]. *S. gordonii* DL1 [28] was cultivated in brain heart infusion broth at 10% CO_2_.

Cultures in the stationary phase were harvested and bacteria suspended in lyophilization solution [10% skin milk with 5% L-Glutamic acid monosodium salt hydrate (Sigma-Aldrich) and 5% dithiothreitol (Sigma-Aldrich)]. Standardized aliquots were lyophilized (FreezoneTriad Freezer Dryers, Labconco, Kansas City, MO, USA) and maintained at −80 °C. For each lot, viability was estimated under appropriate conditions.

On the day of each inoculation, lyophilized bacteria of the microbial consortium were suspended in BHI HM broth, incubated anaerobically for 6 h to achieve a physiological state, and suspended in 2% carboxymethylcellulose gel (LabSynth, São Paulo, Brazil) in PBS to reach 2 × 10^12^ CFU/mL of each strain. Lyophilized *L. acidophilus* LA5 was suspended in 2% carboxymethylcellulose gel in PBS to reach 2 × 10^10^ CFU/mL. Then, bacterial suspensions were immediately inoculated in the mice, with an interval of 4–5 h between the inoculations of the probiotic and the microbial consortium.

### 2.4. Experimental Periodontitis and Probiotic Administration

Detailed procedures were reported previously [24]. Animals received kanamycin (Inlab, São Paulo, Brazil) and amoxicillin (EMS, São Paulo, Brazil) diluted to g/mL in drinking water for four days [28,29], and their oral cavities were rinsed with 2% chlorhexidine digluconate for the last two days [30].

After the two-day wash-out period, the animals of the L+ groups were treated with 50 µL of *L. acidophilus* LA5 containing 1 × 10^9^ CFU and inoculated in the oral cavity with the aid of a gavage needle, once a day, starting on the first day of the experimental period, for 44 days. Animals of the P+ groups received 50 µL of the microbial consortium suspension containing 1 × 10^11^ CFU of each strain by oral gavage, five days/week, totaling 25 inoculations, starting on the second day of the experimental period, and concluding 12 days before euthanasia. Control groups P- and/or L- received only the vehicles of the microbial consortium and/or the probiotic (2% carboxymethylcellulose gel in PBS) for the same volumes and days as the P+ and/or L+ groups.

### 2.5. Sample Collection

At the end of the experimental period (45 days), mice were anesthetized with ketamine/xylazine and euthanized by cervical dislocation. Oral biofilm samples were collected using micro brushes. Gut samples comprised the jejunal microbiota obtained after opening the small intestine with a scalpel and removing its content with a wooden spatula. Samples were placed in TRIS EDTA (TE) buffer (pH 8.0) for microbiome analyses. The right hemi maxilla was transferred to a tube containing 4% formaldehyde solution, maintained for 24 h at RT, and stored at 4 °C in PBS until further analysis.

### 2.6. Microcomputed Tomography (μCT) Analysis

Alveolar bone loss was determined in the right hemimaxilla using a microtomograph (SkyScan 1176, version 1.1, Bruker Biospin, Kontich, Belgian) at 45 kV voltage, 550 µA current, 8.71 µm pixel size, and 0.2 mm aluminum filter. The alignment of three-dimensional (3D) images was performed using the DataViewer (Skyscan) with the occlusal plane oriented parallel to the transverse plane. The region of interest (ROI) for bone volume measurement was set between the distal surface of the first molar and the mesial surface of the second molar, using a 60 × 30-pixel area covering 15 coronal sections starting from the enamel–cementum junction of the second. The analysis was performed using CTAnalyser software Version 1.15.4.0, Skyscan.

### 2.7. Oral and Gut Microbiome Sequencing

DNA was extracted from oral biofilms and small intestine content samples using the MASTER PURE^TM^ DNA PURIFICATION KIT (Epicentre^®^ Illumina Company, Madison, WI, USA). The purified DNA quality was determined using a Qubit 2.0 fluorometer (Thermo-Fisher Scientific, Carlsbad, CA, USA). Negative controls manipulated with the samples were included.

A barcoded primer set based on universal primers Bakt_341F CCTACGGGNGGCWGCAG and Bakt_805R GACTACHVGGGTATCTAATCC [31] was used to amplify the hypervariable V3–V4 region of the *16SrRNA* gene. DNA samples were sequenced by Macrogen (Seoul, Republic of Korea) using the Illumina MiSeq 2 × 250 platform, following the manufacturer’s protocol. Illumina sequencing data were submitted to the Sequence Read Archive (SRA) under BioProject identification number PRJNA994097.

Sequences were analyzed using Qiime 2 2022.8 software [32]. Sequences were demultiplexed, and reads were filtered for quality score (mean q > 10) using Dada 2, quality of phred 10 (90%), and for chimeric sequences. Trimmed sequences were clustered into operational taxonomic units (OTUs) at a similarity threshold of 97% [33]. Taxonomy was assigned using the Silva database (SILVA_SSU database version 132–99, accessed in August 2019). Alpha diversity was calculated for species richness (Chao 1), Pielou, Shannon–Weaver, and Simpson indexes [34], and differences among groups were determined using Kruskal–Wallis with Dunn’s post hoc test. Beta diversity analyses were based on distance matrices of weighted and unweighted Unifrac, Bray Curtis, and Jaccard, and reported according to principal coordinate analysis (PCoA) [32,35]. To determine whether the visually observed differences were statistically significant, PERMANOVA (Permutational Multivariate Analysis of Variance Using Distance Matrices) was performed with 999 permutations. The effects of the different treatments on the microbial mean taxa abundance at the different taxonomic levels of oral and gut communities were evaluated by ANCOM (analysis of composition of microbiomes) [36]. The W value (number of rejections of the null hypothesis assuming that the mean log absolute abundances of all taxa do not differ by the same amount among studied groups) empirical cut-off was set at the 75th percentile [37].

### 2.8. Statistical Analysis

Alveolar bone porosity and volume (%) were tested for normality using the Kolmogorov–Smirnov test with Lilliefors correlation, and homogeneity of variances was assessed by the F test. One-way ANOVA followed by Tukey’s multiple comparison was used to identify inter-group differences. Statistical significance was established at *p* < 0.05. The analyses were performed using the GraphPad Prism^®^ Version 6.0 statistical package (La Jolla, CA, USA).

## 3. Results

### 3.1. Effect of L. acidophilus on Controlling Alveolar Bone Loss

**Figure 1:**
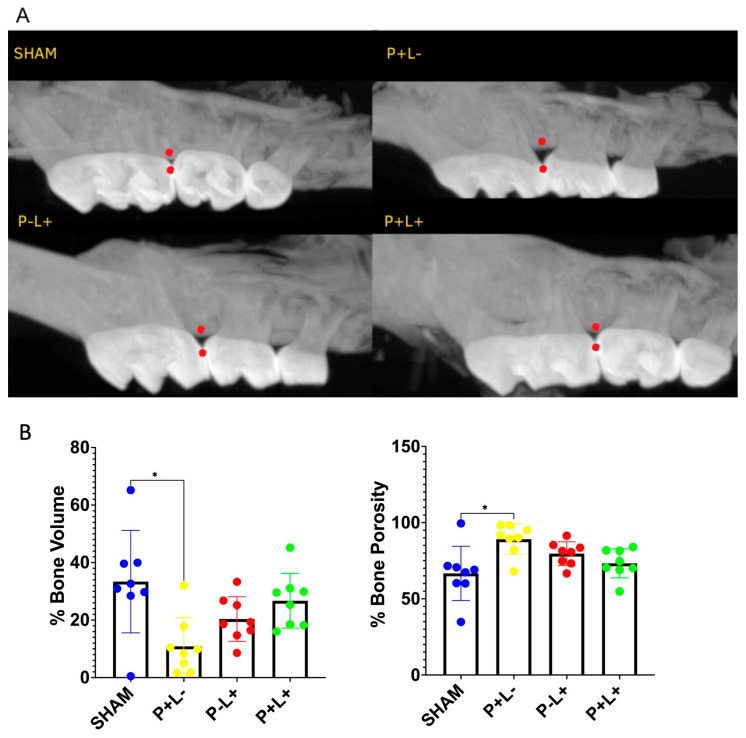
Administration of *L. acidophilus* La5 prevents alveolar bone loss induced by the infection with a microbial consortium. Alveolar bone levels were determined 12 days after twenty-five inoculations of a microbial consortium formed by *P. gingivalis, P. intermedia, F. nucleatum*, and *S. gordonii* (P+ groups) and/or 1 day after 44 days of administration of 1 × 10^9^ CFU LA5 (L+ groups). (**A**): Representative images of alveolar bone. Data were obtained at the region between the red points by Micro-CT analysis of the right hemimaxilla (Pixel size: 8.71 μm). (**B**): % alveolar bone volume (average and sd) and % bone porosity (average and sd), determined in pixels^3^ in C57Bl/6 mice submitted to different treatments: SHAM (negative control), P+L- (positive control, inoculated with the microbial consortium), P-L+ (*L. acidophilus* LA5), P+L+ (microbial consortium + *L. acidophilus* LA5). * Statistically significant difference in relation to the negative control (SHAM). ANOVA, Tukey’s multiple comparison, *p* < 0.05%.

The oral inoculation with the microbial consortium resulted in significant alveolar bone loss as evidenced by decreased bone volume and increased porosity in the P+L- group compared to SHAM (Figure 1). This deleterious effect was attenuated by the treatment with probiotics. While pathogen-inoculated groups presented mean 10.8% (±10.0) bone volume and mean 89.13% (±10.02) porosity in the measured ROI, treatment with *L. acidophilus* LA-5 elevated bone volume to mean 26.7% (±9.52) and decreased bone porosity to mean 73.27% (±9.52). *L. acidophilus* administration (P-L+) per se resulted in minimal and insignificant bone loss.

### 3.2. Effect of L. acidophilus on Reshaping Oral and Gut Microbiomes

A total of 1029,613 (oral) and 1043,808 (gut) raw reads were obtained, of which 901,401 (oral) and 647,954 (gut) reads remained after quality filtering and sequence processing. Although each group comprised eight animals, some samples did not yield enough DNA to enable amplification and sequencing. Thus, the oral samples evaluated were obtained from five animals in the SHAM group, eight for P+L-, six for P-L+, and six for the P+L+ group, and there were the same numbers for the gut samples, except for the P+L+ group, which comprised seven gut samples.

The oral sequences were assigned to 36,056 Amplicon sequence variants (ASVs), with an average of 507.831549 ASVs per sample (range: 189.338028–669.901408). Gut sequences comprised 24,628 ASVs, with an average of 315.459591 ASVs per sample (range: 103.607595–474.291139). Oral samples comprised 432 OTUs, whereas gut samples comprised 493 OTUs. Rarefaction curves indicated that similar sequence depths were achieved for all oral and gut samples.

**Figure 2:**
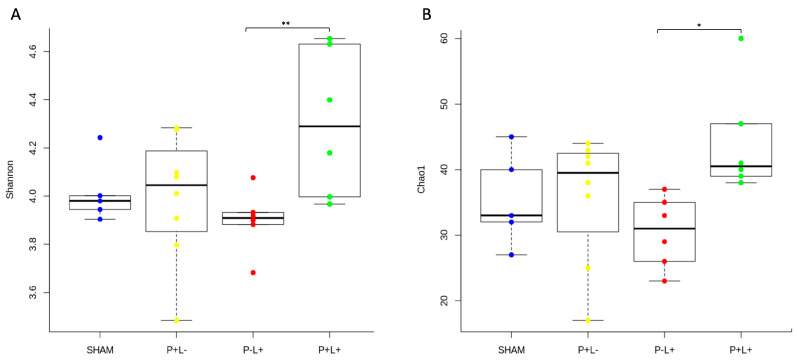
Phylogenetic alpha diversity indices (Chao in (**A**), and Shannon in (**B**)) of the oral microbial communities of C57Bl/6 mice submitted to different treatments: SHAM (negative control), P+L- (positive control, inoculated with the microbial consortium), P-L+ (*L. acidophilus* LA5), P+L+ (microbial consortium + *L. acidophilus* LA5). Kruskal–Wallis with Dunn’s test, significant differences * *p* < 0.05, ** *p* < 0.01.

Administration of the microbial consortium (P+L-) and/or the probiotic (P-L+ and P+L+) did not alter the alpha diversity indexes of oral and gut niches when these groups were compared to the SHAM (P-L-) group. However, the richness and evenness (Chao1 and Shannon diversity indexes, respectively) of the oral microbiome were higher under pathogen infection and probiotic co-treatment (P+L+) than for the P-L+ group (Figure 2A,B). This suggests that the administration of the probiotic in mice infected with the microbial consortium leads to increased diversity in the oral cavity when compared to the administration of the probiotic alone in healthy animals. Alpha diversity indexes did not differ when the gut microbiomes of the four studied groups were compared (Supplemental Figure S1).

**Figure 3:**
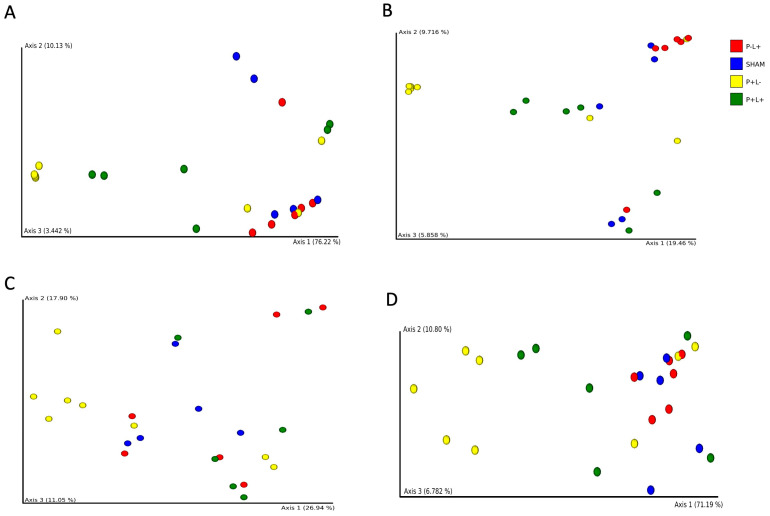
Principal Coordinates Analyses (PCoA) based on Bray–Curtis (**A**), Jaccard (**B**), unweighted (**C**) and weighted (**D**) Unifrac distances of oral microbial communities of C57Bl/6 mice submitted to different treatments: SHAM (negative control), P+L- (positive control, inoculated with the microbial consortium), P-L+ (*L. acidophilus* LA5), P+L+ (microbial consortium + *L. acidophilus* LA5). Each dot represents one sample.

Beta diversity analysis revealed that administration of the microbial consortium did not lead to differences in the structure of the oral microbial communities (SHAM versus P+L-). The administration of the probiotic to non-infected animals (SHAM versus P-L+) did not result in significant differences in the oral microbiome. However, PERMANOVA revealed that the oral microbiomes of P-L+ differed from P+L- by using Bray–Curtis, Jaccard and weighted Unifrac distances (*p* < 0.005). P-L+ and P+L+ also differed by using Bray-Curtis and Jaccard distances (*p* < 0.05), while SHAM differed from P+L+ by using Bray–Curtis distance (*p* < 0.05) (Figure 3).

**Figure 4:**
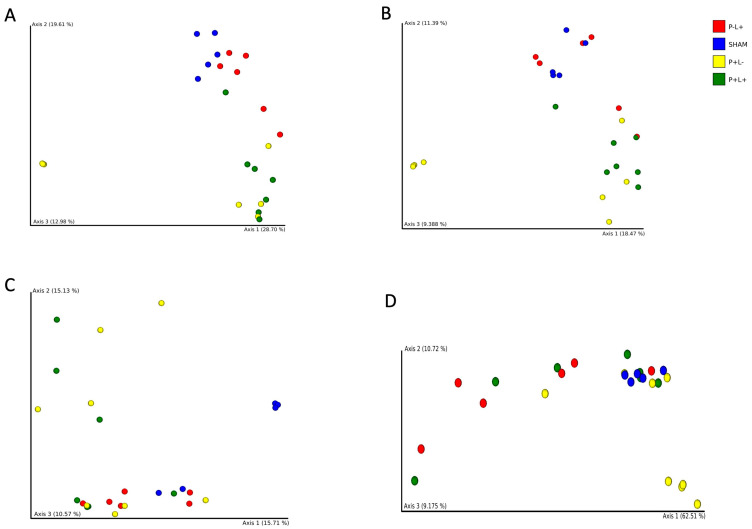
Principal Coordinates Analyses (PCoA) based on Bray–Curtis (**A**), Jaccard (**B**), unweighted (**C**) and weighted (**D**) Unifrac distances of gut microbial communities of C57Bl/6 mice submitted to different treatments: SHAM (negative control), P+L- (positive control, inoculated with the microbial consortium), P-L+ (*L. acidophilus* LA5), P+L+ (microbial consortium + *L. acidophilus* LA5). Each dot represents one sample.

Gut samples from each studied group clustered apart from the others by using Bray–Curtis, Jaccard, unweighted and weighted Unifrac distances, except for P-L+ versus P+L+ by using weighted Unifrac, and P+L- versus P+L+ by using unweighted Unifrac distances. Thus, administration of the probiotic and/or the microbial consortium induced remarkable changes in the gut microbiome, as visualized in PCoA plots (Figure 4).

**Figure 5:**
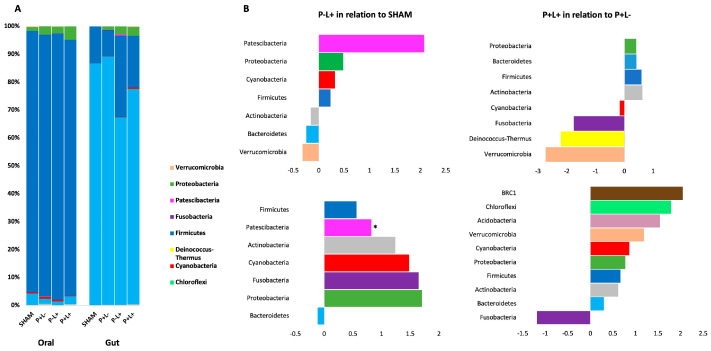
Phyla relative abundance (%)-based plots (in **A**) and fold changes of abundance (in **B**) of oral and gut microbial communities of C57Bl/6 mice submitted to different treatments: SHAM (negative control), P+L- (positive control, inoculated with the microbial consortium), P-L+ (*L. acidophilus* LA5), P+L+ (microbial consortium + *L. acidophilus* LA5). The effect of *L. acidophilus* LA5 in the oral biofilm and gut microbiomes was determined by comparing the abundance of taxons in the P-L+ group and SHAM, and in the P+L+ group and P+L-. * ANCOM, significant differences.

**Figure 6:**
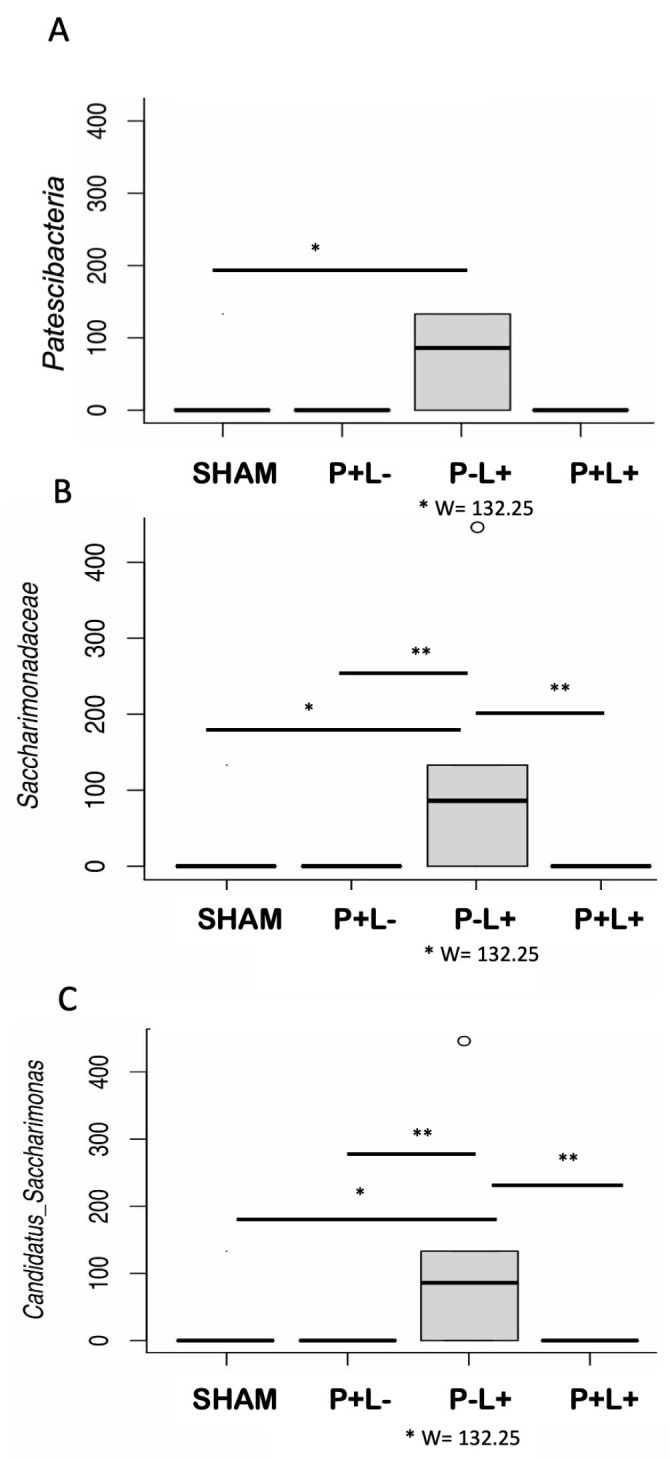
The composition of the gut microbiome differed among the groups regarding the phylum Patescibacteria (in **A**), the family *Saccharimonadaceae* (in **B**), and the genus Candidatus *Saccharimonas* (in **C**) in C57Bl/6 mice submitted to different treatments: SHAM (negative control), P+L- (positive control, inoculated with the microbial consortium), P-L+ (*L. acidophilus* LA5), P+L+ (microbial consortium + *L. acidophilus* LA5). ANCOM revealed differences in microbial mean taxa abundance among the groups. W values for the P-L+ group are shown using the 75th percentile of the W distribution as the empirical cut-off value. Kruskal–Wallis with Dunn’s pos hoc test. Significant differences * *p* < 0.05; ** *p* < 0.01.

Firmicutes was the most abundant phylum in all oral communities, whereas Bacteroidetes was more abundant in gut samples, independently on the treatment (Figure 5A). No differences in abundance at the phylum level among the groups were observed either in oral or gut microbiome analysis, except for an enrichment in the superphylum Patescibacteria in the gut of the P-L+ group (W at 75% = 132.25) (Figure 5B and Figure 6A). However, when the abundance of each phylum was compared as fold changes among groups, we could observe that Patescibacteria was also more abundant in the oral biofilm of mice of the P-L+ group compared to SHAM (Figure 6B). The effect of administration of *L. acidophilus* LA5 to otherwise non-infected animals (P-L- versus SHAM) and infected animals (P+L+ versus P+L-) in fold changes of the abundance of different phyla in the oral biofilm and gut samples is shown Figure 5B. These data (in fold changes) at other taxonomic levels are shown in Supplemental file Figure S2 (Class), Figure S3 (Order), Figure S4 (Family), and Figure S5 (Genus Level).

**Figure 7:**
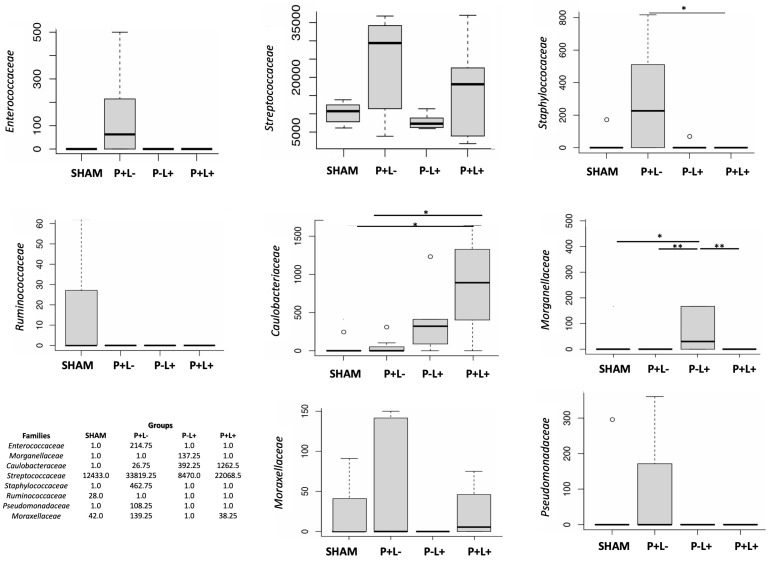
The composition of the oral microbiome differed among the groups at the family level of C57Bl/6 mice submitted to different treatments: SHAM (negative control), P+L- (positive control, inoculated with the microbial consortium), P-L+ (*L. acidophilus* LA5), P+L+ (microbial consortium + *L. acidophilus* LA5). ANCOM revealed differences in microbial mean taxa abundance among the groups. The mean abundance of each family is represented in the graphs. W values are shown in the table using the 75th percentile of the W distribution as the empirical cut-off value. Kruskal–Wallis with Dunn’s pos hoc test. Significant differences * *p* < 0.05; ** *p* < 0.01.

Differences in taxa abundance in the oral microbiome were not seen at high taxonomic levels, but the abundance of several taxa at the family level differed among groups (Figure 7). ANCOM analysis indicated that administration of the microbial consortium increased the abundance of *Enterococcaceae, Streptococcaceae*, *Staphylococcaceae*, *Moraxellaceae*, *Pseudomonadaceae,* and *Caulobacteriaceae* in the oral microbiome, as attested when the P+L- group was compared to SHAM. On the other hand, the administration of *L. acidophilus* LA5 (P-L+) led to the increased abundance of *Caulobacteriaceae* and *Morganellaceae* and the decreased abundance of *Moraxellaceae* when compared to SHAM. All three experimental groups showed reduced abundance of *Ruminococcaceae* when compared to SHAM. More importantly, the administration of *L. acidophilus* LA5 to the animals infected with the microbial consortium (P+L+) abolished most of the differences in the oral microbiome promoted by the microbial consortium by decreasing the abundance of *Enterococcaceae, Pseudomonadaceae, Moraxellaceae*, *Staphylococcacea*, and *Streptococcaceae*, reaching abundance levels similar to those demonstrated in the SHAM group. However, the levels of *Caulobacteriaceae* continued to increase with the co-administration of *L. acidophilus* LA5 to the infected group (Figure 7 and Figure S4). The gut microbiome of P-L+ mice differed from the other groups due to an increased abundance of the family *Saccharimonadaceae*, genus *Saccharimonas* (Figure 6B,C), although other differences could be observed at the family level when data were converted to fold changes (Figure S4).

## 4. Discussion

We aimed to evaluate the potential of the probiotic strain *L. acidophilus* La5 to control periodontal destruction and to shape the resident microbiome in a model of periodontal destruction induced by a microbial consortium associated with periodontitis in humans. The experimental model employed was able to induce alveolar bone loss in infected mice and to promote a shift in the abundance of certain organisms in the oral and gut microbiomes. The use of the probiotic, on the other side, was effective in modulating such deleterious effects.

Although a microbial consortium was orally inoculated in mice in order to achieve a synergistic effect, as seen in periodontitis [38], the main pathogen implicated in periodontitis, *P. gingivalis,* was not detected in the oral biofilm at the end of the experimental period. However, the altered oral and gut microbiomes persisted for at least 12 days after the last inoculation of the pathogens. Our results are similar to data in which mice were experimentally monoinfected with *P. gingivalis*, oral dysbiosis was seen after 10 days of the last inoculation of the pathogen, and *P. gingivalis* was detected in very low levels in few of the inoculated animals [39].

The inoculation of the consortium did not lead to changes in diversity indexes, possibly due to the previous use of antimicrobials to clear the resident microbiota [40]. However, the dental biofilms of mice infected with the microbial consortium leading to alveolar bone loss were characterized by increased abundance of the families *Enterococcaceae, Streptococcaceae, Staphylococccaceae, Pseudomonadaceae, Caulobacteriaceae*, and *Moraxellaceae* and decreased levels of *Ruminococcaceae* when compared to SHAM infected animals. Previous studies also reported that *P. gingivalis* induces overgrowth of the genera *Staphylococcus* and *Streptococcus* [39], and families *Caulobacteriaceae, Moraxellaceae*, and *Staphylococcace* [40] in the oral cavity of mice. However, increased levels of *Pseudomonadaceae* were elicited exclusively by the microbial consortium, although our data differ from others [39,40] also on the oral sampling site (gingival tissue versus dental biofilm). The microbial consortium also exerted an effect on the structure of the gut microbiome, as evidenced by Beta diversity analysis, where the gut microbiome of SHAM clustered apart from the P+L- group, although no specific taxa were consistently more or less abundant in the infected group when compared to SHAM by using ANCOM.

The administration of *L. acidophilus* La5 to the infected group was able to attenuate alveolar bone loss induced by the microbial consortium. This beneficial effect was accompanied by a microbial shift in the oral microbiome, where the abundance of *Enterococcaceae, Streptococcaceae, Staphylococccaceae, Pseudomonadaceae,* and *Moraxellaceae* returned to levels observed in the oral microbiome of SHAM infected animals. However, treatment with *L. acidophilus* LA5 promoted an enrichment of *Caulobacteriaceae* in the oral microbiome of animals infected with the microbial consortium. The effect of increased levels of *Caulobacteriaceae* in the oral cavity has not been established, but most members of this family are commensals with few exceptions in patients with underlying conditions [40].

The oral intake of *L. acidophilus* La5 was also able to induce changes in the jejunal microbiome of the mice, with increased levels of the superphylum Patescibacteria and the family *Saccharimonadaceae,* genus *Candidatus Saccharomonas*, but these alterations were lost in mice that were infected with the microbial consortium and received concomitant administration of *L. acidophilus* La5. Little is known on the role of the superphylum Patescibacteria, a group of still-uncultivated bacteria [41] reported in low abundance in fecal samples of mice [42]. In humans, its increased abundance in stool samples was associated with multiple sclerosis in children [43], whereas its decreased abundance was reported in the saliva of subjects with brain tumors [44]. On the other hand, the increased abundance of family *Sacchararomonadaceae*, genus *Candidatus Saccharomonas* in the gut after the administration of *L. acidophilus* LA5 in otherwise non-infected mice may be beneficial to the host. The enrichment of these SCFA (short chain fatty acids)-producing bacteria leads to increased serum acetate concentrations, which is implicated in the regulation of glucose metabolism [45]. Furthermore, the abundance of *Candidatus Saccharomonas* in the gut of mice is negatively correlated with levels of pro-inflammatory cytokines in serum [46].

*L. acidophilus* is a member of the human resident microbiota and can be found in the oral cavity, gastrointestinal tract, and vaginal mucosa [47]. However, the oral administration of *L. acidophilus* LA5 in mice, using a similar protocol as used in the current study, did not yield the detection of the species *L. acidophilus* in the oral nor in the gut microbiomes [22]. Long lasting colonization by probiotics is not expected either in the oral cavity or in the gut [48,49], indicating the need for probiotic usage on a daily basis for effective results, as performed in the present study.

These results should be taken under the limitations of the present experimental model, where exogenous strains of human origin were inoculated in young mice with unbalanced microbiomes disturbed by the previous use of chlorhexidine and antimicrobials. Other studies should be performed in order to assess the effect of *L. acidophilus* LA5 on the control of periodontal and systemic inflammation, since this strain is known to provide benefits in inflammatory diseases [14,15,16,17].

Our data suggest that *L. acidophilus* LA5 can lead to shifts in unbalanced oral and gut microbiomes, leading to the control of periodontitis.

## 5. Conclusions

The oral administration of *L. acidophilus* LA5 can attenuate bone resorption and revert most of the shifts in the oral microbiome induced by infection with a microbial consortium of human periodontal pathogens. Further studies are still needed to elucidate the mechanisms underlying the beneficial properties of *L. acidophilus* LA5. Nevertheless, our data suggest that this strain is a promising candidate as an adjuvant to the periodontal treatment, used either alone or in combination with other probiotic strains.

## Data Availability

Sequencing data were submitted to the Sequence Read Archive (SRA) under BioProject identification number PRJNA994097.

## Supplementary Materials

The following supporting information can be downloaded at: https://www.mdpi.com/article/10.3390/microorganisms12061057/s1, Figure S1. Phylogenetic Alpha diversity indices (Chao in A, and Simpson in B) of the gut microbial communities of C57Bl/6 mice submitted to different treatments: SHAM (negative control), P+L- (positive control, inoculated with the microbial consortium), P-L+ (*L. acidophilus* LA5), P+L+ (microbial consortium + *L. acidophilus* LA5). Kruskal Wallis with Dunn´s pos hoc test. No significant differences, *p* > 0.05. Figure S2: Fold changes of abundance at the Class taxonomic level of oral and gut microbial communities of C57Bl/6 mice submitted to different treatments: SHAM (negative control), P+L- (positive control, inoculated with the microbial consortium), P-L+ (*L. acidophilus* LA5), P+L+ (microbial consortium + *L. acidophilus* LA5). The effect of *L. acidophilus* LA5 in the oral biofilm and gut microbiomes was determined by comparing the abundance of taxons in P-L+ group and SHAM, and in P+L+ group and P+L-. Data reported as fold changes of abundance. Figure S3: Fold changes of abundance at the Order taxonomic level of oral and gut microbial communities of C57Bl/6 mice submitted to different treatments: SHAM (negative control), P+L- (positive control, inoculated with the microbial consortium), P-L+ (*L. acidophilus* LA5), P+L+ (microbial consortium + *L. acidophilus* LA5). The effect of *L. acidophilus* LA5 in the oral biofilm and gut microbiomes was determined by comparing the abundance of taxons in P-L+ group and SHAM, and in P+L+ group and P+L-. Data reported as fold changes of abundance. Figure S4: Fold changes of abundance at the family taxonomic level of oral and gut microbial communities of C57Bl/6 mice submitted to different treatments: SHAM (negative control), P+L- (positive control, inoculated with the microbial consortium), P-L+ (*L. acidophilus* LA5), P+L+ (microbial consortium + *L. acidophilus* LA5). The effect of *L. acidophilus* LA5 in the oral biofilm and gut microbiomes was determined by comparing the abundance of taxons in P-L+ group and SHAM, and in P+L+ group and P+L-. Data reported as fold changes of abundance. * ANCOM, significant differences. Figure S5: Fold changes of abundance at the genus taxonomic level of oral and gut microbial communities of C57Bl/6 mice submitted to different treatments: SHAM (negative control), P+L- (positive control, inoculated with the microbial consortium), P-L+ (*L. acidophilus* LA5), P+L+ (microbial consortium + *L. acidophilus* LA5). The effect of *L. acidophilus* LA5 in the oral biofilm and gut microbiomes was determined by comparing the abundance of taxons in P-L+ group and SHAM, and in P+L+ group and P+L-. Data reported as fold changes of abundance.

## References

[1] Hajishengallis G., Lamont R.J. (2012). Beyond the Red Complex and into More Complexity: The Polymicrobial Synergy and Dysbiosis (PSD) Model of Periodontal Disease Etiology. Mol. Oral Microbiol..

[2] Amado P.P.P., Kawamoto D., Albuquerque-Souza E., Franco D.C., Saraiva L., Casarin R.C.V., Horliana A.C.R.T., Mayer M.P.A. (2020). Oral and Fecal Microbiome in Molar-Incisor Pattern Periodontitis. Front. Cell Infect. Microbiol..

[3] Arimatsu K., Yamada H., Miyazawa H., Minagawa T., Nakajima M., Ryder M.I., Gotoh K., Motooka D., Nakamura S., Iida T. (2014). Oral Pathobiont Induces Systemic Inflammation and Metabolic Changes Associated with Alteration of Gut Microbiota. Sci. Rep..

[4] Kawamoto D., Borges R., Ribeiro R.A., de Souza R.F., Amado P.P.P., Saraiva L., Horliana A.C.R.T., Faveri M., Mayer M.P.A. (2021). Oral Dysbiosis in Severe Forms of Periodontitis Is Associated with Gut Dysbiosis and Correlated with Salivary Inflammatory Mediators: A Preliminary Study. Front. Oral Health.

[5] Hill C., Guarner F., Reid G., Gibson G.R., Merenstein D.J., Pot B., Morelli L., Canani R.B., Flint H.J., Salminen S. (2014). The International Scientific Association for Probiotics and Prebiotics Consensus Statement on the Scope and Appropriate Use of the Term Probiotic. Nat. Rev. Gastroenterol. Hepatol..

[6] Raff A., Hunt L.C. (2012). Probiotics for Periodontal Health: A Review of the Literature. J. Dent. Hyg..

[7] Gomes A.C., Bueno A.A., de Souza R.G.M., Mota J.F. (2014). Gut Microbiota, Probiotics and Diabetes. Nutr. J..

[8] Mousquer C.R., Della Bona A., Milani D.C., Callegari-Jacques S.M., Ishikawa K., Mayer M.P.A., Rösing C.K., Fornari F. (2020). Are *Lactobacillus salivarius* G60 and Inulin More Efficacious to Treat Patients with Oral Halitosis and Tongue Coating than the Probiotic Alone and Placebo? A Randomized Clinical Trial. J. Periodontol..

[9] Wolowczuk I., Verwaerde C., Viltart O., Delanoye A., Delacre M., Pot B., Grangette C. (2008). Feeding Our Immune System: Impact on Metabolism. Clin. Dev. Immunol..

[10] Laleman I., Pauwels M., Quirynen M., Teughels W. (2020). A Dual-strain *Lactobacilli reuteri* Probiotic Improves the Treatment of Residual Pockets: A Randomized Controlled Clinical Trial. J. Clin. Periodontol..

[11] Alanzi A., Honkala S., Honkala E., Varghese A., Tolvanen M., Söderling E. (2018). Effect of *Lactobacillus rhamnosus* and *Bifidobacterium lactis* on Gingival Health, Dental Plaque, and Periodontopathogens in Adolescents: A Randomised Placebo-Controlled Clinical Trial. Benef. Microbes.

[12] Gheisary Z., Mahmood R., Harri Shivanantham A., Liu J., Lieffers J.R.L., Papagerakis P., Papagerakis S. (2022). The Clinical, Microbiological, and Immunological Effects of Probiotic Supplementation on Prevention and Treatment of Periodontal Diseases: A Systematic Review and Meta-Analysis. Nutrients.

[13] Li J., Zhao G., Zhang H.M., Zhu F.F. (2023). Probiotic Adjuvant Treatment in Combination with Scaling and Root Planing in Chronic Periodontitis: A Systematic Review and Meta-Analysis. Benef. Microbes.

[14] Asgharian H., Homayouni-Rad A., Mirghafourvand M., Mohammad-Alizadeh-Charandabi S. (2020). Effect of Probiotic Yoghurt on Plasma Glucose in Overweight and Obese Pregnant Women: A Randomized Controlled Clinical Trial. Eur. J. Nutr..

[15] Rezazadeh L., Gargari B.P., Jafarabadi M.A., Alipour B. (2019). Effects of Probiotic Yogurt on Glycemic Indexes and Endothelial Dysfunction Markers in Patients with Metabolic Syndrome. Nutrition.

[16] Zarrati M., Shidfar F., Nourijelyani K., Mofid V., Hossein Zadeh-Attar M.J., Bidad K., Najafi F., Gheflati Z., Chamari M., Salehi E. (2013). *Lactobacillus acidophilus* La5, Bifidobacterium BB12, and *Lactobacillus casei* DN001 Modulate Gene Expression of Subset Specific Transcription Factors and Cytokines in Peripheral Blood Mononuclear Cells of Obese and Overweight People. BioFactors.

[17] Nabavi S., Rafraf M., Somi M.H., Homayouni-Rad A., Asghari-Jafarabadi M. (2014). Effects of Probiotic Yogurt Consumption on Metabolic Factors in Individuals with Nonalcoholic Fatty Liver Disease. J. Dairy Sci..

[18] Albuquerque-Souza E., Balzarini D., Ando-Suguimoto E.S., Ishikawa K.H., Simionato M.R.L., Holzhausen M., Mayer M.P.A. (2019). Probiotics Alter the Immune Response of Gingival Epithelial Cells Challenged by *Porphyromonas gingivalis*. J. Periodontal Res..

[19] Bueno M.R., Ishikawa K.H., Almeida-Santos G., Ando-Suguimoto E.S., Shimabukuro N., Kawamoto D., Mayer M.P.A. (2022). Lactobacilli Attenuate the Effect of *Aggregatibacter actinomycetemcomitans* Infection in Gingival Epithelial Cells. Front. Microbiol..

[20] Ishikawa K.H., Mita D., Kawamoto D., Nicoli J.R., Albuquerque-Souza E., Lorenzetti Simionato M.R., Mayer M.P.A. (2020). Probiotics Alter Biofilm Formation and the Transcription of *Porphyromonas gingivalis* Virulence-Associated Genes. J. Oral Microbiol..

[21] Ishikawa K.H., Bueno M.R., Kawamoto D., Simionato M.R.L., Mayer M.P.A. (2021). Lactobacilli Postbiotics Reduce Biofilm Formation and Alter Transcription of Virulence Genes of *Aggregatibacter actinomycetemcomitans*. Mol. Oral Microbiol..

[22] Bueno M.R., Martins F.H., Rocha C.M., Kawamoto D., Ishikawa K.H., Ando-Suguimoto E.S., Carlucci A.R., Arroteia L.S., Casarin R.V., Mayer M.P.A. (2024). *Lactobacillus acidophilus* LA-5 Ameliorates Inflammation and Alveolar Bone Loss Promoted by *A. actinomycetemcomitans* and *S. gordonii* in Mice and Impacts Oral and Gut Microbiomes. Microorganisms.

[23] Medina M., Izquierdo E., Ennahar S., Sanz Y. (2007). Differential Immunomodulatory Properties of *Bifidobacterium logum* Strains: Relevance to Probiotic Selection and Clinical Applications. Clin. Exp. Immunol..

[24] Shimabukuro N., Cataruci A.C.d.S., Ishikawa K.H., de Oliveira B.E., Kawamoto D., Ando-Suguimoto E.S., Albuquerque-Souza E., Nicoli J.R., Ferreira C.M., de Lima J. (2021). Bifidobacterium Strains Present Distinct Effects on the Control of Alveolar Bone Loss in a Periodontitis Experimental Model. Front. Pharmacol..

[25] Charan J., Kantharia N.D. (2013). How to Calculate Sample Size in Animal Studies?. J. Pharmacol. Pharmacother..

[26] Barbosa G.M., Colombo A.V., Rodrigues P.H., Simionato M.R.L. (2015). Intraspecies Variability Affects Heterotypic Biofilms of *Porphyromonas gingivalis* and *Prevotella intermedia*: Evidences of Strain-Dependence Biofilm Modulation by Physical Contact and by Released Soluble Factors. PLoS ONE.

[27] Rickard A.H., Palmer R.J., Blehert D.S., Campagna S.R., Semmelhack M.F., Egland P.G., Bassler B.L., Kolenbrander P.E. (2006). Autoinducer 2: A Concentration-dependent Signal for Mutualistic Bacterial Biofilm Growth. Mol. Microbiol..

[28] Pakula R. (1965). Factors Regulating Competence in Transformation of Streptococci. J. Bacteriol..

[29] Gatej S.M., Marino V., Bright R., Fitzsimmons T.R., Gully N., Zilm P., Gibson R.J., Edwards S., Bartold P.M. (2018). Probiotic *Lactobacillus rhamnosus GG* Prevents Alveolar Bone Loss in a Mouse Model of Experimental Periodontitis. J. Clin. Periodontol..

[30] Kang J., de Brito Bezerra B., Pacios S., Andriankaja O., Li Y., Tsiagbe V., Schreiner H., Fine D.H., Graves D.T. (2012). *Aggregatibacter actinomycetemcomitans* Infection Enhances Apoptosis In Vivo through a Caspase-3-Dependent Mechanism in Experimental Periodontitis. Infect. Immun..

[31] Herlemann D.P., Labrenz M., Jürgens K., Bertilsson S., Waniek J.J., Andersson A.F. (2011). Transitions in Bacterial Communities along the 2000 Km Salinity Gradient of the Baltic Sea. ISME J..

[32] Bolyen E., Rideout J.R., Dillon M.R., Bokulich N.A., Abnet C.C., Al-Ghalith G.A., Alexander H., Alm E.J., Arumugam M., Asnicar F. (2019). Reproducible, Interactive, Scalable and Extensible Microbiome Data Science Using QIIME 2. Nat. Biotechnol..

[33] Caporaso J.G., Kuczynski J., Stombaugh J., Bittinger K., Bushman F.D., Costello E.K., Fierer N., Peña A.G., Goodrich J.K., Gordon J.I. (2010). QIIME Allows Analysis of High-Throughput Community Sequencing Data. Nat. Methods.

[34] Kim B.-R., Shin J., Guevarra R.B., Lee J.H., Kim D.W., Seol K.-H., Lee J.-H., Kim H.B., Isaacson R.E. (2017). Deciphering Diversity Indices for a Better Understanding of Microbial Communities. J. Microbiol. Biotechnol..

[35] Lozupone C., Lladser M.E., Knights D., Stombaugh J., Knight R. (2011). UniFrac: An Effective Distance Metric for Microbial Community Comparison. ISME J..

[36] Mandal S., Van Treuren W., White R.A., Eggesbø M., Knight R., Peddada S.D. (2015). Analysis of Composition of Microbiomes: A Novel Method for Studying Microbial Composition. Microb. Ecol. Health Dis..

[37] Lin H., Peddada S. (2020). Das Analysis of Compositions of Microbiomes with Bias Correction. Nat. Commun..

[38] Hajishengallis G. (2014). Immunomicrobial Pathogenesis of Periodontitis: Keystones, Pathobionts, and Host Response. Trends Immunol..

[39] Payne M.A., Hashim A., Alsam A., Joseph S., Aduse-Opoku J., Wade W.G., Curtis M.A. (2019). Horizontal and Vertical Transfer of Oral Microbial Dysbiosis and Periodontal Disease. J. Dent. Res..

[40] Simas A.M., Kramer C.D., Weinberg E.O., Genco C.A. (2021). Oral Infection with a Periodontal Pathogen Alters Oral and Gut Microbiomes. Anaerobe.

[41] Tian R., Ning D., He Z., Zhang P., Spencer S.J., Gao S., Shi W., Wu L., Zhang Y., Yang Y. (2020). Small and Mighty: Adaptation of Superphylum *Patescibacteria* to Groundwater Environment Drives Their Genome Simplicity. Microbiome.

[42] Lu L., Tang M., Li J., Xie Y., Li Y., Xie J., Zhou L., Liu Y., Yu X. (2021). Gut Microbiota and Serum Metabolic Signatures of High-Fat-Induced Bone Loss in Mice. Front. Cell Infect. Microbiol..

[43] Tremlett H., Zhu F., Arnold D., Bar-Or A., Bernstein C.N., Bonner C., Forbes J.D., Graham M., Hart J., Knox N.C. (2021). The Gut Microbiota in Pediatric Multiple Sclerosis and Demyelinating Syndromes. Ann. Clin. Transl. Neurol..

[44] Wen Y., Feng L., Wang H., Zhou H., Li Q., Zhang W., Wang M., Li Y., Luan X., Jiang Z. (2021). Association Between Oral Microbiota and Human Brain Glioma Grade: A Case-Control Study. Front. Microbiol..

[45] Luo L., Luo J., Cai Y., Fu M., Li W., Shi L., Liu J., Dong R., Xu X., Tu L. (2022). Inulin-Type Fructans Change the Gut Microbiota and Prevent the Development of Diabetic Nephropathy. Pharmacol. Res..

[46] Wang J., Xiang Q., Gu S., Gu Y., Yao M., Huang W., Gao W., Tang L.-L. (2022). Short- and Long-Term Effects of Different Antibiotics on the Gut Microbiota and Cytokines Level in Mice. Infect. Drug Resist..

[47] Bull M., Plummer S., Marchesi J., Mahenthiralingam E. (2013). The Life History of *Lactobacillus acidophilus* as a Probiotic: A Tale of Revisionary Taxonomy, Misidentification and Commercial Success. FEMS Microbiol. Lett..

[48] Yli-Knuuttila H., Snäll J., Kari K., Meurman J.H. (2006). Colonization of *Lactobacillus rhamnosus* GG in the Oral Cavity. Oral Microbiol. Immunol..

[49] Collado M., Isolauri E., Salminen S., Sanz Y. (2009). The Impact of Probiotic on Gut Health. Curr. Drug Metab..

