# Supplementary material for: Oral Administration of Lactobacillus acidophilus LA5 Prevents Alveolar Bone Loss and Alters Oral and Gut Microbiomes in a Murine Periodontitis Experimental Model
Source: Microorganisms. 2024 May 24;12(6):1057. doi: 10.3390/microorganisms12061057 (PMC11205731; doi:10.3390/microorganisms12061057)
Supplement: Supplementary file 1 [file microorganisms-12-01057-s001.zip › microorganisms-2971381-supplementary.pdf]

Supplemental File

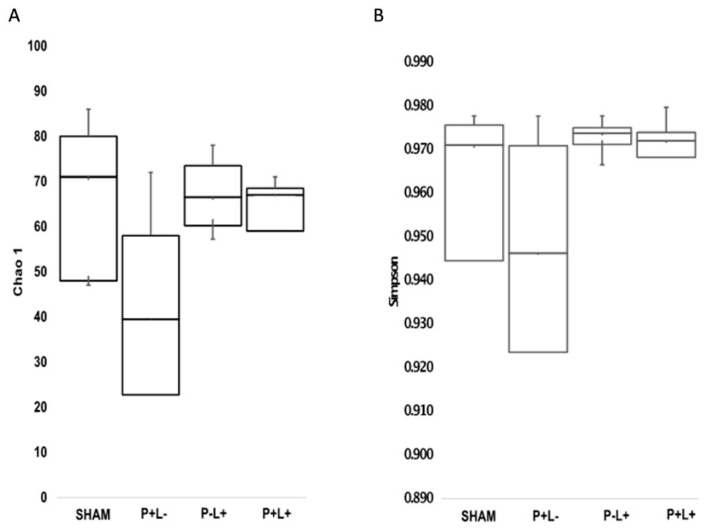

Figure S1. Phylogenetic Alpha diversity indices (Chao in A, and Simpson in B) of the gut microbial communities of C57Bl/6 mice submitted to different treatments: SHAM (negative control), P+L- (positive control, inoculated with the microbial consortium), P-L+ (*L. acidophilus* LA5), P+L+ (microbial consortium + *L. acidophilus* LA5). Kruskal Wallis with Dunn´s pos hoc test. No significant differences, $p>0.05$.

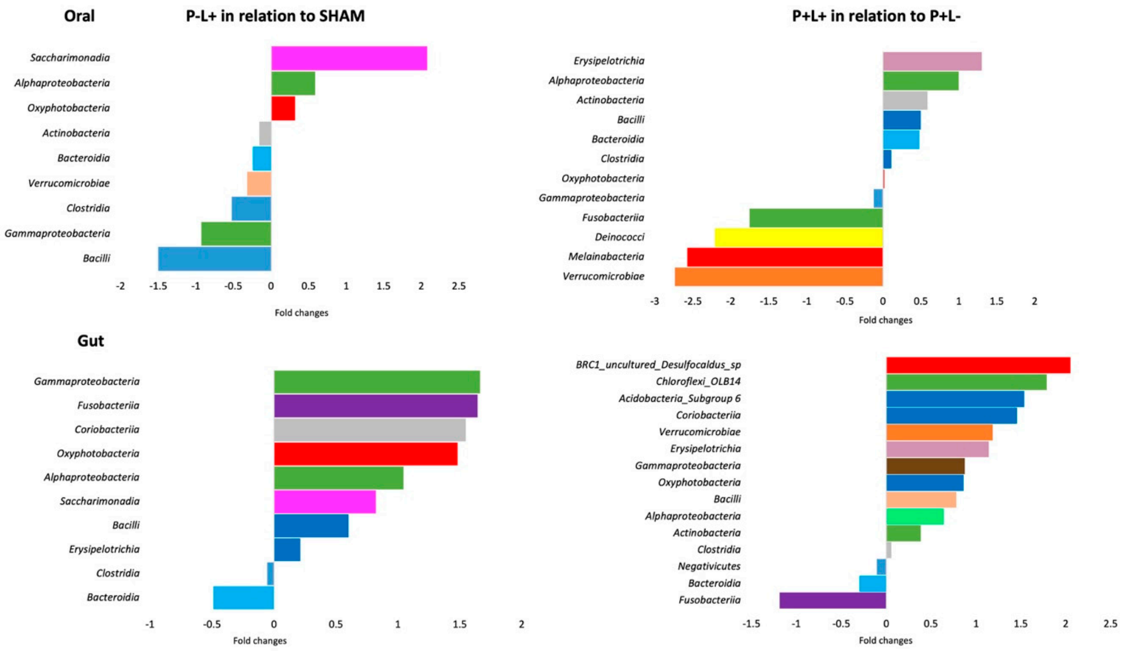

Figure S2. Fold changes of abundance at the Class taxonomic level of oral and gut microbial communities of C57Bl/6 mice submitted to different treatments: SHAM (negative control), P+L- (positive control, inoculated with the microbial consortium), P-L+ (*L. acidophilus* LA5), P+L+ (microbial consortium + *L. acidophilus* LA5). The effect of *L.acidophilus* LA5 in the oral biofilm and gut microbiomes was determined by comparing the abundance of taxons in P-L+ group and SHAM, and in P+L+ group and P+L-. Data reported as fold changes of abundance.

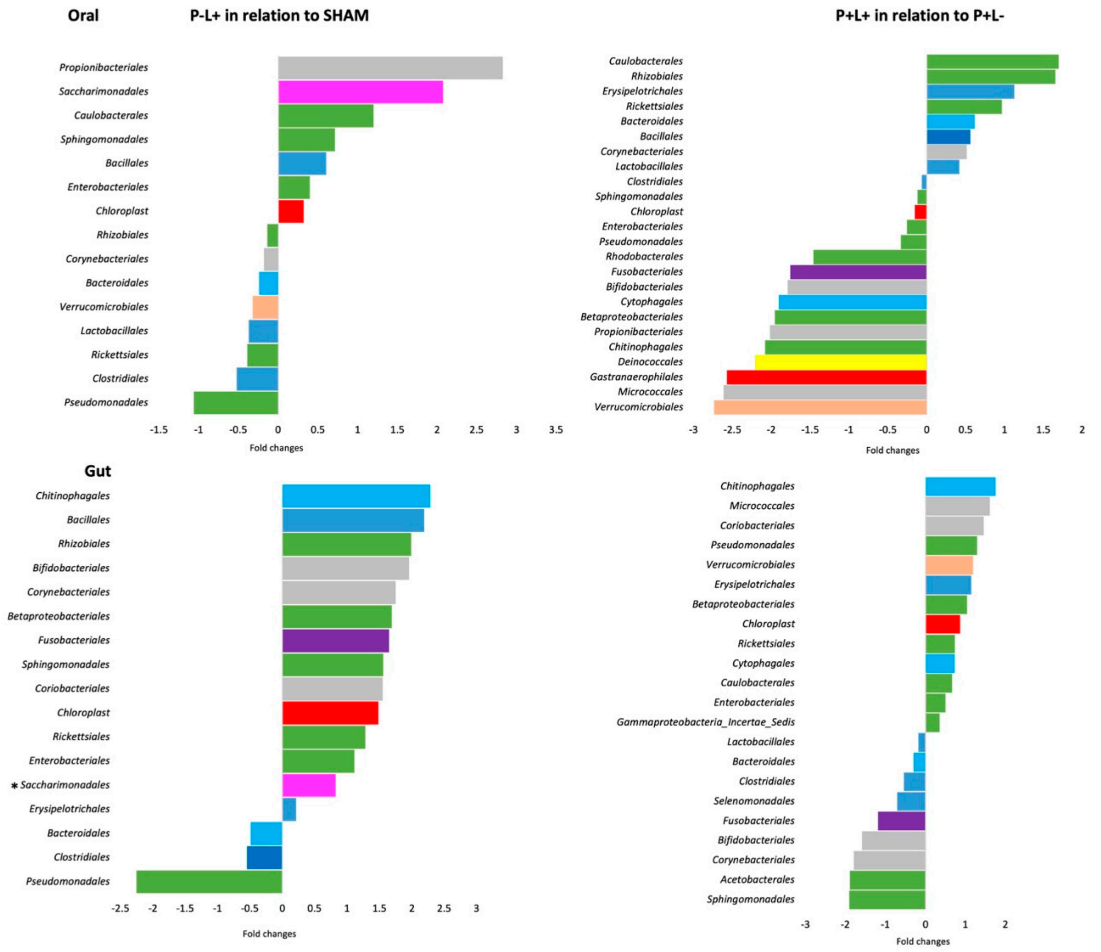

Figure S3. Fold changes of abundance at the Order taxonomic level of oral and gut microbial communities of C57Bl/6 mice submitted to different treatments: SHAM (negative control), P+L- (positive control, inoculated with the microbial consortium), P-L+ (*L. acidophilus* LA5), P+L+ (microbial consortium + *L. acidophilus* LA5). The effect of *L.acidophilus* LA5 in the oral biofilm and gut microbiomes was determined by comparing the abundance of taxons in P-L+ group and SHAM, and in P+L+ group and P+L-. Data reported as fold changes of abundance.

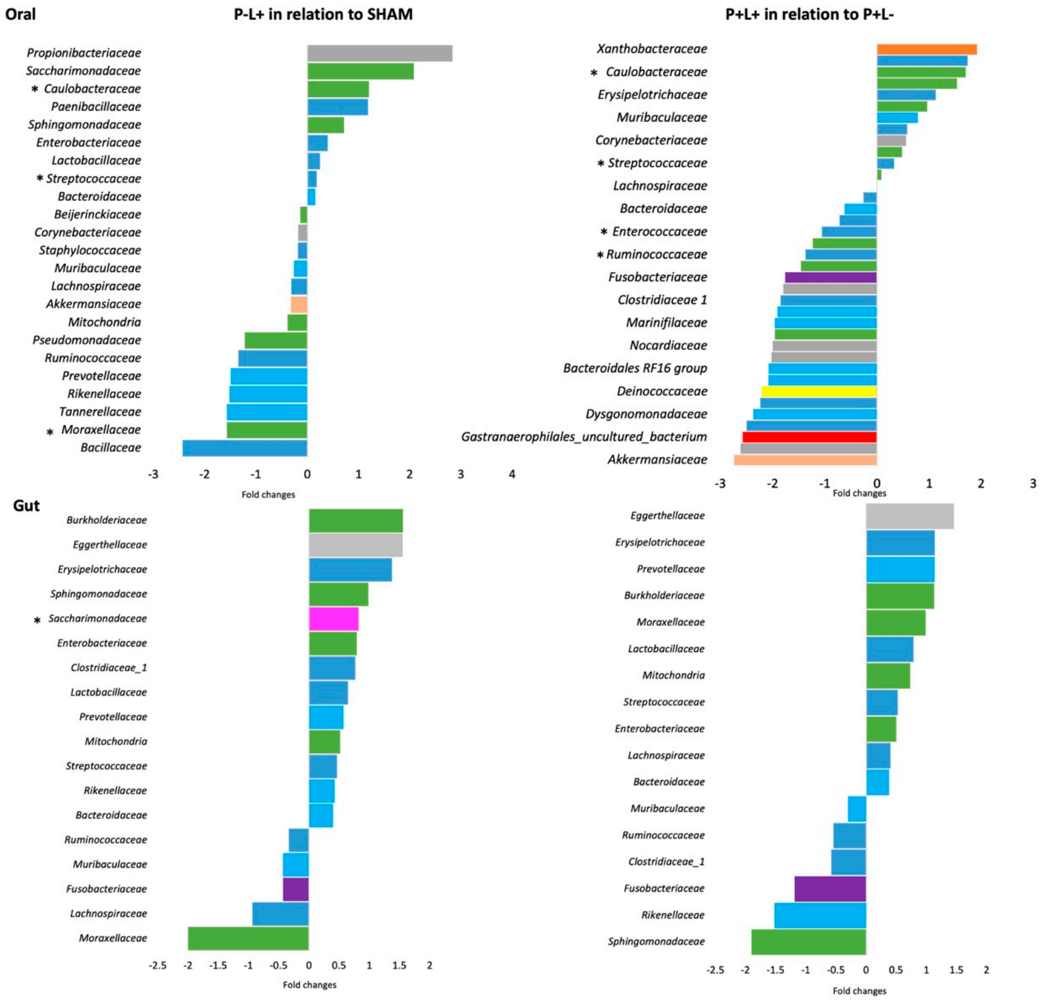

Figure S4. Fold changes of abundance at the family taxonomic level of oral and gut microbial communities of C57Bl/6 mice submitted to different treatments: SHAM (negative control), P+L- (positive control, inoculated with the microbial consortium), P-L+ (*L. acidophilus* LA5), P+L+ (microbial consortium + *L. acidophilus* LA5). The effect of *L.acidophilus* LA5 in the oral biofilm and gut microbiomes was determined by comparing the abundance of taxons in P-L+ group and SHAM, and in P+L+ group and P+L-. Data reported as fold changes of abundance. * ANCOM, significant differences.

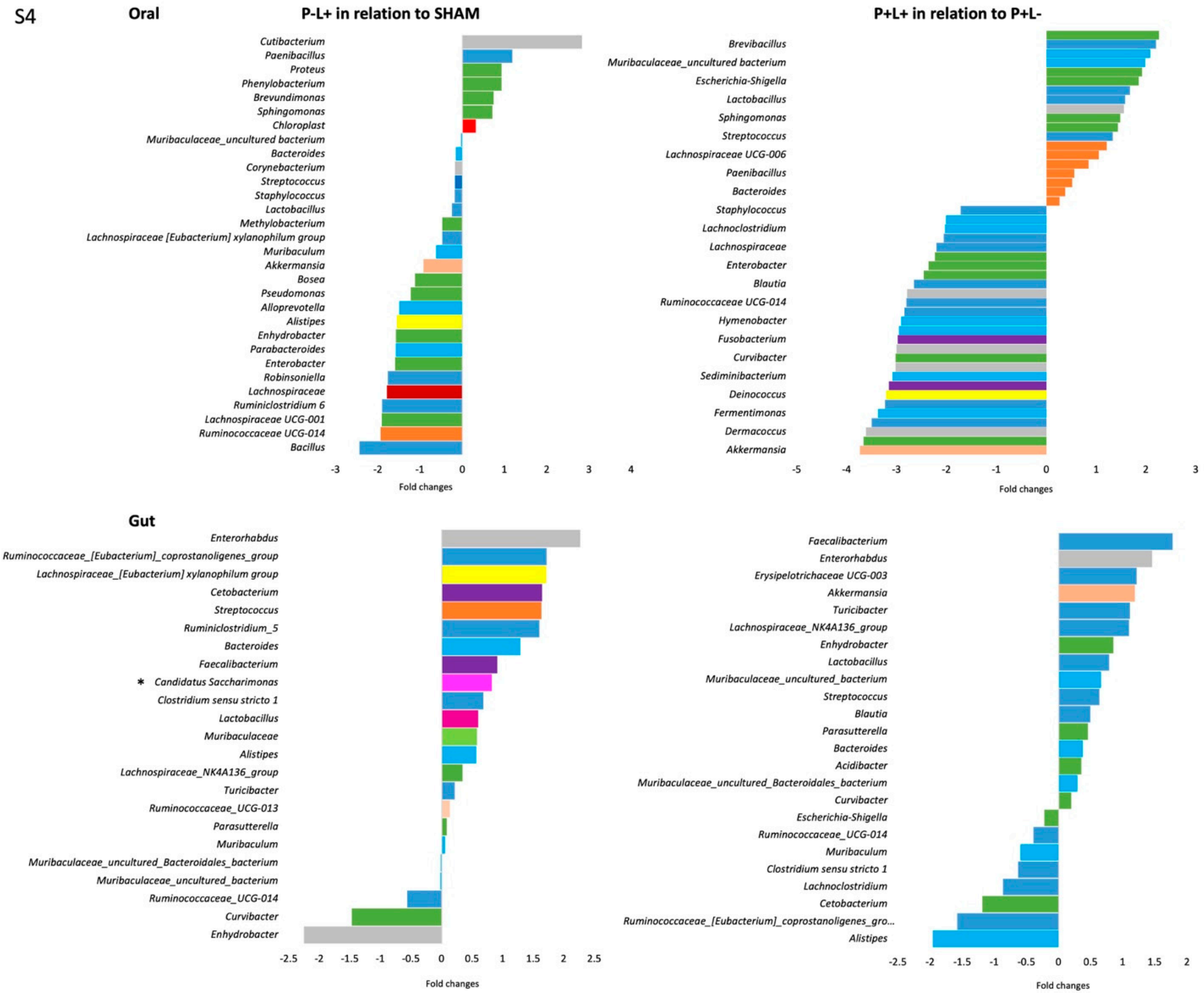

Figure S5. Fold changes of abundance at the genus taxonomic level of oral and gut microbial communities of C57Bl/6 mice submitted to different treatments: SHAM (negative control), P+L- (positive control, inoculated with the microbial consortium), P-L+ (*L. acidophilus* LA5), P+L+ (microbial consortium + *L. acidophilus* LA5). The effect of *L.acidophilus* LA5 in the oral biofilm and gut microbiomes was determined by comparing the abundance of taxons in P-L+ group and SHAM, and in P+L+ group and P+L-. Data reported as fold changes of abundance.
